# PROMISE‑MINO: A Prospective, Open‑Label, Multicenter, Single‑Arm, Phase IV Study of the Safety, Local Tolerability, and Efficacy of Topical Minocycline Hydrochloride 4% Gel in Indian Patients With Moderate‑to‑Severe Acne Vulgaris

**DOI:** 10.7759/cureus.101599

**Published:** 2026-01-15

**Authors:** Vishalakshi Viswanath, Swetalina Pradhan, Mamta Bhura, Richa Gupta, Prerna Suryatale, H Banguru, Abhishek De, Vijay Sonkar, Sumit Bhushan, Rujuta Gadkari, Dhiraj Dhoot, Ashwin Balasubramanian, Saiprasad Patil, Hanmant Barkate

**Affiliations:** 1 Department of Dermatology, Rajiv Gandhi Medical College (RGMC) Hospital, Thane, IND; 2 Department of Dermatology, All India Institute of Medical Sciences, Patna, IND; 3 Department of Dermatology, New Leelamani Hospital, Kanpur, IND; 4 Department of Dermatology, Aatman Hospital, Ahmedabad, IND; 5 Department of Dermatology, Jeevan Rekha Hospital, Belgaum, IND; 6 Department of Dermatology, Krishna Rajendra Hospital, Mysore, IND; 7 Department of Dermatology, Wizderm Speciality Skin and Hair Clinic, Kolkata, IND; 8 Department of Dermatology, Sarojini Naidu Medical College, Agra, IND; 9 Department of Global Medical Affairs, Glenmark Pharmaceuticals Limited, Mumbai, IND

**Keywords:** acne, efficacy, gel, india, minocycline 4%, phase 4, phase iv, safety, topical

## Abstract

Background/objectives: With increasing resistance to clindamycin, minocycline shows low resistance rates in *Cutibacterium acnes*. Topical minocycline 4% foam was approved in the USA (2019) and as a gel formulation in India (2022) for moderate-to-severe acne vulgaris (AV). However, there is a paucity of clinical data regarding the use of topical minocycline in acne. This study was conducted to assess the safety, tolerability, and efficacy of topical minocycline 4% gel in Indian patients.

Methods: This prospective, open-label, multicenter, non-comparative, 12-week phase IV study included 256 patients aged ≥9 years with moderate-to-severe acne vulgaris and excluded those with other facial dermatological conditions. All patients received topical minocycline 4% gel once daily for 12 weeks. The primary endpoints were the absolute local skin tolerability scores at week 12. The secondary endpoints included adverse events (AEs), changes in mean inflammatory and non-inflammatory lesion counts, and Investigator Global Assessment (IGA) treatment success at week 12 from baseline.

Results: Of 256 patients, 242 completed the study. A total of 100 adverse events (AEs) were reported in 65 patients (25.39%), of which 53 events in 42 (16.41%) were drug-related, with the majority being mild to moderate. These AEs were related to local tolerability and included pruritus (n = 26, 10.16%), erythema (n = 20, 7.81%), skin hyperpigmentation (n = 18, 7.03%), dry skin (n = 17, 6.64%), and exfoliation (n = 8, 3.13%). Reductions of 78% in inflammatory and 74% in non‑inflammatory acne lesions were observed at week 12, and 64.4% of patients achieved IGA treatment success.

Conclusions: Topical minocycline 4% gel showed an acceptable safety and tolerability profile with no new safety signals. Additionally, there was a clinically significant reduction in both inflammatory and non-inflammatory lesion counts, as well as a good IGA treatment success rate. Thus, it can be considered as an alternative for managing moderate-to-severe acne vulgaris.

## Introduction

Acne vulgaris (AV) is the most prevalent dermatological condition worldwide, affecting approximately 85% of adolescents and often persisting into adulthood, thereby impacting nearly all age groups [1-4]. Mild acne is typically managed with topical treatments, whereas moderate-to-severe acne often requires systemic antibiotics, with tetracyclines being the primary choice [5-7]. However, as a chronic condition, acne frequently necessitates prolonged treatment [8].

The American Academy of Dermatology (AAD) recommends oral minocycline and doxycycline as first-line therapies for moderate-to-severe acne [2,9]. Despite their efficacy, these drugs have significant systemic adverse effects. Antibiotic resistance poses a challenge to dermatological practice. Among the available antibiotics, tetracyclines, particularly minocycline, exhibit the lowest resistance rates, whereas increasing resistance has been observed with topical macrolides and lincosamides such as clindamycin and erythromycin, leading to the reduced use of these agents [1,9].

To reduce systemic exposure while retaining efficacy against *Cutibacterium acnes *(*C. acnes*), topical minocycline 4% was developed [9]. Topical minocycline 4% foam was approved in 2019 in the USA by the regulator [10]. This topical formulation offered similar efficacy against *C. acnes* but minimal systemic adverse effects as compared to oral minocycline. Long-term data are available for up to 52 weeks, suggesting that topical minocycline 4% foam is well tolerated in terms of local and systemic adverse effects and has good efficacy in moderate-to-severe acne management [11].

In India, owing to thermostability concerns, the Drug Controller General of India (DCGI) did not approve the foam formulation. Glenmark Pharmaceuticals Limited, recognizing the need, obtained marketing authorization for topical minocycline 4% gel in moderate-to-severe acne for patients nine years and above, with the regulatory condition to conduct a phase IV clinical trial [12]. Therefore, this study was conducted with the primary objective being safety and local skin tolerability, and the secondary objective being efficacy in Indian patients with moderate-to-severe acne vulgaris.

## Materials and methods

Study design

This prospective, open-label, multicenter, single-arm, 12-week phase IV study was conducted across eight sites in India from April 2023 to May 2024. The study design was in accordance with regulatory requirements. The principles of the Declaration of Helsinki and Good Clinical Practices as per the guidelines of the Indian regulator and the International Council of Harmonization were followed during the study. Ethics committee approval was obtained for each study site. Written informed consent was obtained from all patients before they were included, and the study was registered in the Clinical Trial Registry of India (CTRI/2023/04/051390).

Participant eligibility criteria

Male or female patients aged nine years or older, having Fitzpatrick skin type III-V, having moderate-to-severe facial acne vulgaris, defined as “20-50 inflammatory lesions (papules, pustules, or nodules), 25-100 non-inflammatory lesions (open and closed comedones), no more than two nodules on the face, and an Investigator Global Assessment (IGA) score of grade 3 (moderate) or grade 4 (severe)” participated in the study [13]. Patients participating in the study were advised not to use concomitant acne medication during the 12-week study period. Patients with any dermatological condition of the face (e.g., acne conglobata, acne fulminans, or facial dermatological conditions that could interfere with clinical evaluations) were excluded.

Interventions

Topical minocycline 4% gel was applied by patients once daily for 12 weeks at approximately the same time of day, preferably at night time one hour before bedtime, to prevent photosensitivity or any related conditions. A standard moisturizer with SPF 30 was provided for use during the daytime, along with appropriate instructions. The use of other anti-acne medications was prohibited. Patient compliance was monitored using standard patient diaries.

Clinical evaluations and endpoints

The primary endpoint was the local skin tolerability at week 12, assessed using absolute scores for erythema, dryness, hyperpigmentation, skin peeling, and pruritus, along with severity, using a 4-point scale (0 = none to 3 = severe). Secondary endpoints included the following: (1) the number of adverse events (AEs), serious adverse events (SAEs), treatment-emergent adverse events (TEAEs), and AEs leading to dose modification/discontinuation of treatment for the duration of the study; (2) changes from baseline in inflammatory and non-inflammatory lesion counts at weeks 3, 6, 9, and 12; and (3) Investigator Global Assessment (IGA) treatment success (dichotomized as yes/no) at weeks 3, 6, 9, and 12. IGA treatment success was defined as an IGA score of 0 or 1 and at least a 2-grade improvement (decrease) from baseline at weeks 3, 6, 9, and 12. An investigator Global Assessment (IGA) was also used to assess acne severity on a 6-point scale, where “0 = clear (skin with no inflammatory or non-inflammatory lesions), 1 = almost clear (rare non-inflammatory lesions with more than one small inflammatory lesion), 2 = mild (greater than grade 1; some non-inflammatory lesions with no more than few inflammatory lesions (papules/pustules only, no nodular lesions)), 3 = moderate (greater than grade 2; up to many non-inflammatory lesions and may have some inflammatory lesions, but no more than one small nodular lesion), 4 = severe (greater than grade 3; up to many non-inflammatory and inflammatory lesions, but no more than a few nodular lesions), and 5 = very severe (≥grade 4; up to many non-inflammatory, inflammatory, and nodules, a few cysts)” [13].

Statistical analysis

According to the binomial and Poisson distributions, if the adverse events (AEs) have a probability of occurrence of 1.3%, the sample size would be 230 in order for the investigators to have a 95% chance of observing at least one case of AE. Considering a 10% dropout, 256 patients were enrolled in the study. Analyses were conducted on an observed‑case basis (i.e., no imputation). All safety analyses were performed using the safety analysis set (SAF). The safety data were summarized descriptively. Efficacy analysis was performed on the intention-to-treat (ITT) population using a descriptive method, with an associated 95% confidence interval (CI) calculated for the efficacy assessments. Quantitative data are presented as mean, median, standard deviation (SD), and range. Qualitative data are presented as frequencies and proportions. The efficacy parameters were compared with the baseline value for the mean change in values using Student’s paired t-test. Two-tailed p-values (<0.05) were considered statistically significant. In the absence of pre‑study correlation estimates between baseline and follow‑up lesion counts, a mid‑range correlation (r = 0.5) was assumed to compute the SD of paired differences and paired t‑statistics. No imputation methods (e.g., last observation carried forward (LOCF)) were used. All statistical analyses were performed using Statistical Analysis System (SAS) version 9.4 (SAS Inc., Cary, NC).

## Results

Baseline characteristics and participant disposition

**Figure 1:**
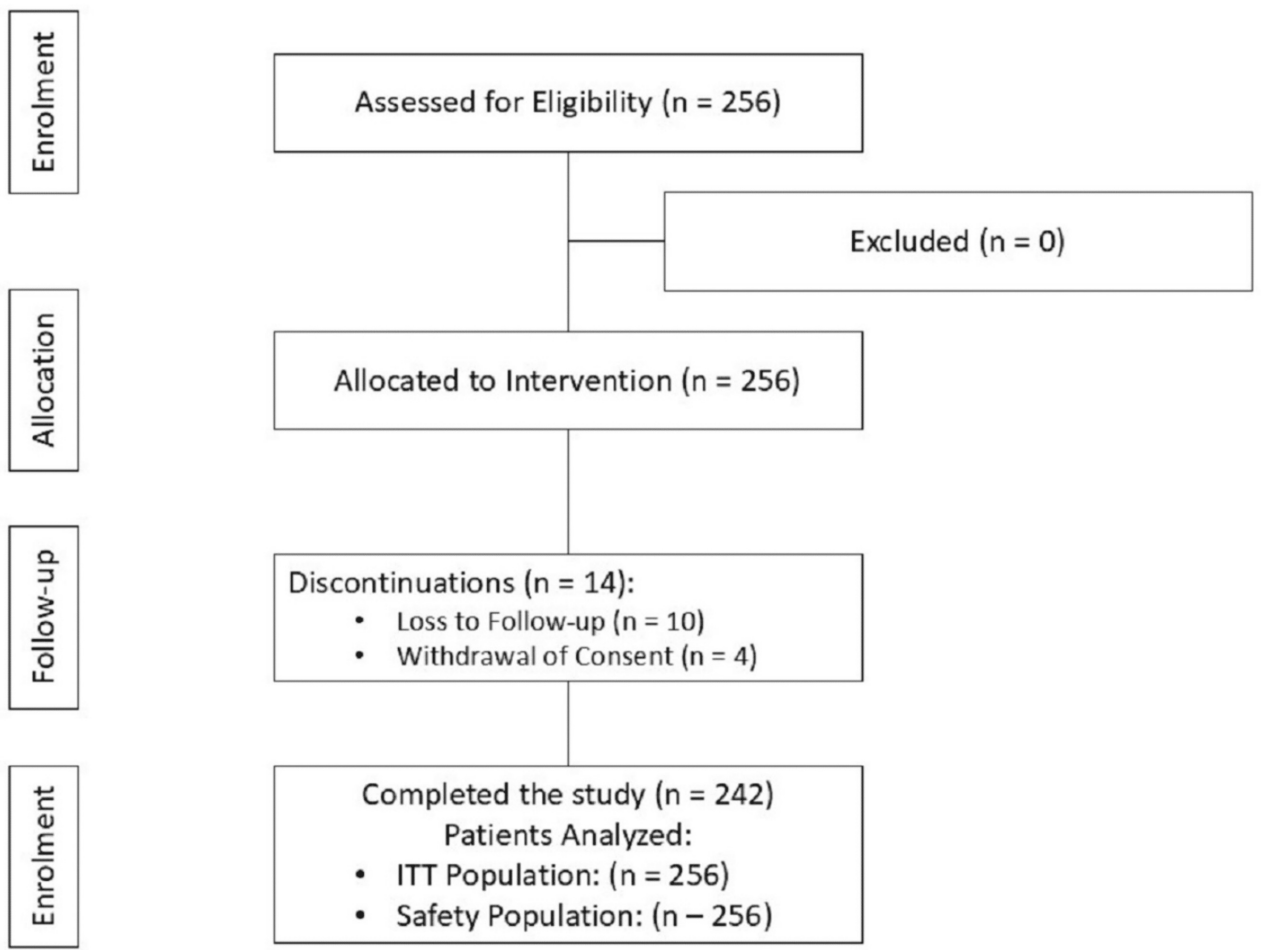
Consort Diagram for Flow of Participants in the Study ITT population: all patients who were included in the study Safety population: all patients who received at least one dose of the investigational product ITT: intention-to-treat

A total of 256 patients were included in the intention-to-treat (ITT) population, with all patients receiving at least one dose of the study drug and followed up for 12 weeks. Of them, 242 (94.53%) completed the study. Fourteen patients (5.47%) discontinued the study because of loss to follow-up or withdrawal of consent. The complete methodology is depicted in Figure 1.

The baseline characteristics of the patients enrolled in the study are presented in Table 1.

**Table 1:** Summary of Demographics and Baseline Characteristics (N = 256) SD: standard deviation

Vital parameter	Value
Mean age (years) ± SD	22 ± 5.14
Female	132 (51.56%)
Male	124 (48.44 %)
Mean height (cm) ± SD	162 ± 8.63
Mean weight (kg) ± SD	57.3 ± 9.55
Mean inflammatory lesion count ± SD	29.81 ± 7.27
Mean non-inflammatory lesion count ± SD	44.07 ± 16.43

Safety and tolerability

A total of 100 treatment-emergent adverse events (TEAEs) were reported in 65 patients (25.39%) (Table 2).

**Table 2:** Overall Summary of Adverse Events: Safety Population (N = 256) AE: adverse event, SAE: serious adverse event, TEAE: treatment-emergent adverse event

Type of adverse event	Number (%)	Number of events
TEAEs	65 (25.39%)	100
Drug-related TEAEs	42 (16.41%)	53
Severe TEAEs	1 (0.39 %)	1
Not related to intervention	27 (10.55%)	47

Most TEAEs were mild to moderate in severity, with no serious adverse events or deaths reported during the study period. Drug‑related TEAEs occurred in 42 patients (16.41%), and one case of severe pruritus (0.39%) was reported; all adverse events were transient and resolved without sequelae.

Hyperpigmentation was predominantly mild to moderate and clinically consistent with post‑inflammatory hyperpigmentation, a commonly observed and expected outcome in patients with acne, particularly in darker skin phototypes. Importantly, no new or progressive pigmentary safety signals were identified over the 12‑week treatment period.

The most commonly reported TEAEs were pruritus (10.16%), erythema (7.81%), skin hyperpigmentation (7.03%), dry skin (6.64%), and skin exfoliation (3.13%) (Table 2). Fewer events of burning sensation and skin discoloration were reported (<1% each).

Local skin tolerability assessments demonstrated a marked improvement by week 12 compared with baseline, supporting favorable local tolerability with continued use (Table 3).

**Table 3:** Summary and Analysis of Skin Tolerability Assessment at Week 12 Compared to Baseline (N = 256)

Symptoms	Visit	Absent (number (%))	Mild (number (%))	Moderate (number (%))	Severe (number (%))
Erythema	Baseline	112 (43.75%)	69 (26.95%)	47 (18.36%)	28 (10.94%)
	Week 12	238 (98.34%)	3 (1.23%)	1 (0.41%)	0 (0%)
Dryness	Baseline	128 (50.00%)	75 (29.30%)	39 (15.23%)	14 (5.47%)
	Week 12	241 (99.6%)	1 (0.41%)	0 (0%)	0 (0%)
Hyperpigmentation	Baseline	71 (27.73%)	113 (44.14%)	67 (26.17%)	5 (1.95%)
	Week 12	212 (87.6%)	17 (7.02%)	12 (4.95%)	1 (0.41%)
Skin peeling	Baseline	175 (68.36%)	60 (23.44%)	19 (7.42%)	2 (0.78%)
	Week 12	242 (100%)	0 (0%)	0 (0%)	0 (0%)
Itching	Baseline	151 (58.98%)	72 (28.13%)	25 (9.77%)	8 (3.13%)
	Week 12	242 (100%)	0 (0%)	0 (0%)	0 (0%)

Efficacy

The mean inflammatory lesion count decreased from 29.81 ± 7.27 at baseline to 6.42 ± 6.63 at week 12, representing an absolute reduction of 23.39 (78%). Reductions were observed as early as week 3 (-7.01) and progressed through week 6 (-13.71) and week 9 (-18.97), with all reductions statistically significant compared with baseline (p < 0.001).

Similarly, the mean non‑inflammatory lesion count decreased from 44.07 ± 16.43 at baseline to 11.44 ± 10.88 at week 12, corresponding to an absolute reduction of 32.63 (74%). Progressive reductions were noted at week 3 (-8.63), week 6 (-18.14), and week 9 (-25.72), with all changes statistically significant versus baseline (p < 0.001) (Table 4).

**Table 4:** Change in Inflammatory and Non-inflammatory Acne Lesions Over the Weeks Compared to Baseline (N = 256) *Paired t-tests compare each visit to baseline within each lesion type; SD of paired differences was imputed with r = 0.5; df = 255; two-sided p-values. SD: standard deviation

Visit	Mean ± SD	t-statistic	p-value*
Inflammatory lesion count (paired t-test, r = 0.5, n = 256, df = 255)			
Baseline	29.81 ± 7.27	-	-
Week 3	22.80 ± 7.88	14.77	<0.001
Week 6	16.10 ± 7.37	29.97	<0.001
Week 9	10.84 ± 6.49	43.91	<0.001
Week 12	6.42 ± 6.63	53.68	<0.001
Non-inflammatory lesion count (paired t-test, r = 0.5, n = 256, df = 255)			
Baseline	44.07 ± 16.43	-	-
Week 3	35.44 ± 14.88	8.79	<0.001
Week 6	25.93 ± 12.55	19.51	<0.001
Week 9	18.35 ± 10.97	28.39	<0.001
Week 12	11.44 ± 10.88	36.06	<0.001

Consistent with these reductions in lesion counts, Investigator Global Assessment (IGA) treatment success increased progressively over time. Success was achieved by 0.39% of patients at week 3, 6.34% at week 6, 24.49% at week 9, and 64.46% at week 12, indicating clinically meaningful improvement with continued treatment.

**Figure 2:**
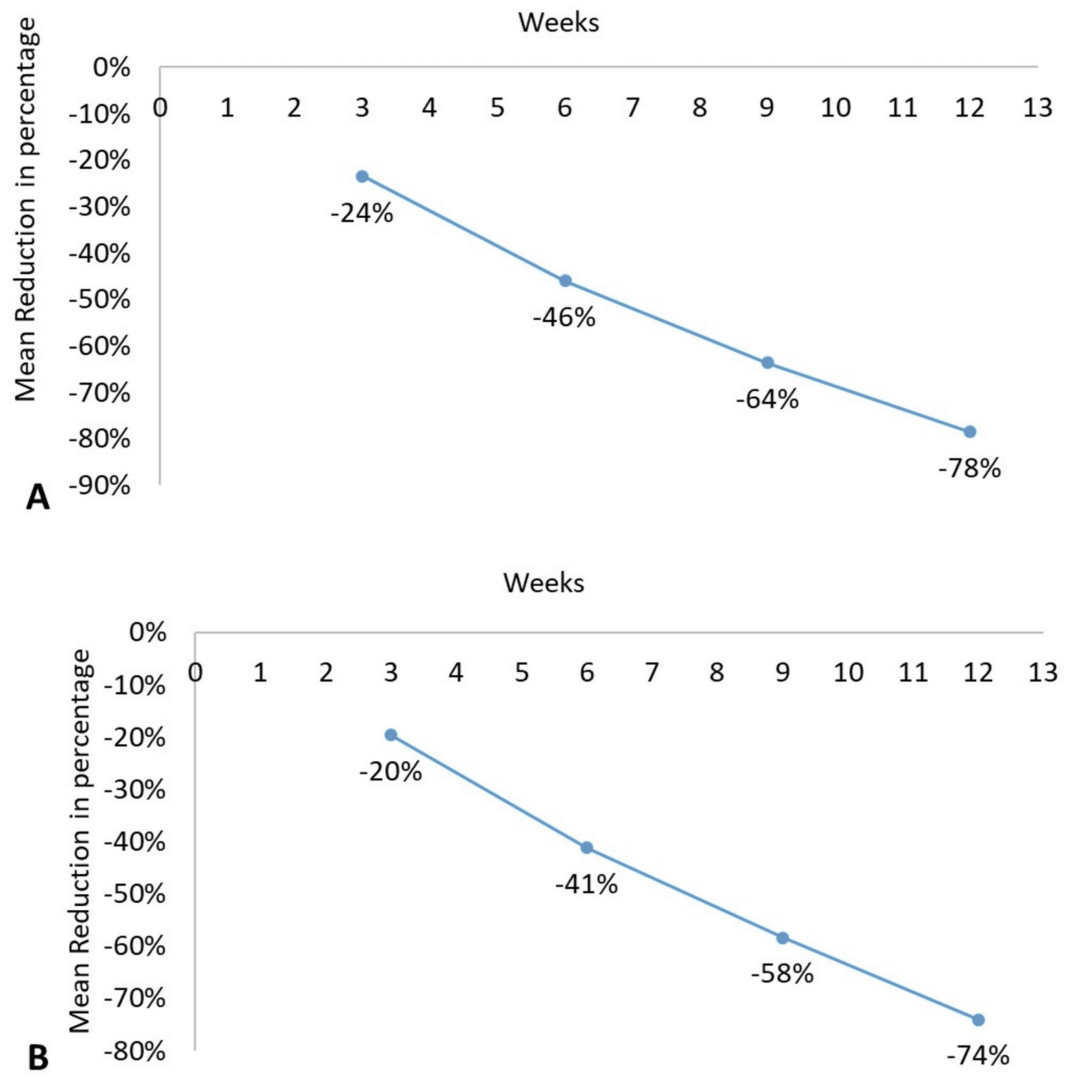
Acne Vulgaris: Percentage Reduction in (A) Inflammatory and (B) Non-inflammatory Lesion Count From Baseline by Visit

The percentage reduction in inflammatory and non-inflammatory lesions is depicted in Figure 2.

## Discussion

The safety, tolerability, and efficacy of topical minocycline 4% gel for the treatment of moderate-to-severe acne were evaluated in a phase IV study involving 256 patients. The results demonstrated that the gel, used for up to 12 weeks, was generally safe and well-tolerated, with few treatment-emergent adverse events (TEAEs). These findings are consistent with those reported in landmark clinical trials of topical minocycline 4% foam formulation, dubbed Study 04 and 05, which enrolled 466 and 495 patients with moderate-to-severe AV, respectively [11,14]. Regarding efficacy, the gel significantly reduced inflammatory and non-inflammatory lesion counts and improved overall disease severity, as assessed using the Investigator Global Assessment (IGA).

The safety outcomes of this phase IV study closely aligned with those of previous landmark clinical trials. In phase III trials dubbed Study 04 and 05, 26.2% of patients reported TEAEs, compared to 25.39% in the current study [14]. Similarly, long-term extension studies reported TEAEs in 22.9% and 32.2% of patients in Study 04 and Study 05, respectively, where most of the adverse events were mild to moderate in severity, with 0.3% and 1.2% of patients experiencing serious adverse events (SAEs) in the topical minocycline group and 0% and 1.2% of the patients experiencing SAEs in the vehicle groups in Study 04 and Study 05, respectively [11]. Across all studies, most TEAEs were mild to moderate and unrelated to treatment.

A key finding of the present study was the absence of severe adverse events commonly associated with oral minocycline, such as fatigue, dizziness, lupus-like syndrome, photosensitivity, and skin pigmentation [15,16]. While pseudotumor cerebri, linked to oral minocycline in adolescents and adults, can present with headache and blurred vision [17], only one patient (0.4%) in the current study reported a mild headache, unrelated to treatment, which did not lead to study discontinuation. It resolved spontaneously and did not warrant further investigation or imaging.

The overall safety profile of the topical minocycline 4% gel was favorable. Furthermore, none of the patients discontinued the study because of adverse events. Dermal tolerability assessments at week 12 showed that over 95% of patients reported no or mild symptoms at the treatment site. Hyperpigmentation (12.4%) was the most common symptom, followed by erythema (1.6%), both of which were mild. Hyperpigmentation was attributed to inflammatory and post-inflammatory acne changes rather than cutaneous discoloration caused by long-term tetracycline use [11]. These findings were similar to the tolerability assessments in the pooled analysis of the phase III trials of topical minocycline 4% foam with hyperpigmentation (11.2%) and erythema (6.2%) [9].

Low adherence to acne therapy, often attributed to delayed clinical responses, dissatisfaction with treatment, or the occurrence of adverse events, is a common challenge [8,18]. In this study, outcomes such as favorable tolerability of the gel, significant reduction in lesion counts, and steady IGA improvement likely contributed to better adherence. Inflammatory and non-inflammatory lesion counts decreased by 78.4% and 74%, respectively, while IGA treatment success was observed in 64.46% of patients. While direct comparisons are limited by the open‑label, non‑comparative design of the present study and differences in formulation and populations, the magnitude of improvement observed here (e.g., lesion count reductions and 64.46% IGA success at week 12) is at least comparable to, and in some metrics greater than, reported outcomes from phase III topical minocycline foam trials. There was a statistically significant reduction in the mean lesion counts in the investigational groups compared to the vehicle groups as early as week 3. IGA treatment success was seen only in 8.09% and 14.66% patients in the topical minocycline group, as compared to 4.77% and 7.89% in the vehicle groups in Study 04 and Study 05, respectively [9]. Overall, topical minocycline 4% gel was found to be a safe, well-tolerated, and effective treatment option for moderate-to-severe acne vulgaris (AV) in Indian patients in the present study. However, the open-label, non-comparative nature of this study was a limitation. While there is only one comparative study between topical minocycline and clindamycin in Indian patients, it favors minocycline over clindamycin [19]. The short duration of study and the lack of microbiological resistance assessment are also limitations. Further comparative and longer studies are warranted to strengthen these findings. Also, microbiological resistance assessment should be included in future studies, which would help with stewardship.

## Conclusions

The results of this phase IV study showed that topical minocycline 4% gel had an acceptable safety and tolerability profile in patients with moderate-to-severe acne vulgaris. In addition, there was a significant reduction in inflammatory and non-inflammatory lesion counts, along with an improvement in the success of IGA treatment. These findings provide real-world phase IV evidence further supporting the use of topical minocycline 4% gel as a therapeutic option for the management of moderate‑to‑severe acne vulgaris in Indian patients.
